# ZnO Nanoparticles Affect *Bacillus subtilis* Cell Growth and Biofilm Formation

**DOI:** 10.1371/journal.pone.0128457

**Published:** 2015-06-03

**Authors:** Yi-Huang Hsueh, Wan-Ju Ke, Chien-Te Hsieh, Kuen-Song Lin, Dong-Ying Tzou, Chao-Lung Chiang

**Affiliations:** 1 Graduate School of Biotechnology and Bioengineering, Yuan Ze University, Taoyuan, Taiwan; 2 Graduate Institute of Biomedical Sciences, and Research Center for Bacterial Pathogenesis, Chang Gung University, Taoyuan, Taiwan; 3 Department of Chemical Engineering and Materials Science, Yuan Ze University, Taoyuan, Taiwan; University Hospital of the Albert-Ludwigs-University Freiburg, GERMANY

## Abstract

Zinc oxide nanoparticles (ZnO NPs) are an important antimicrobial additive in many industrial applications. However, mass-produced ZnO NPs are ultimately disposed of in the environment, which can threaten soil-dwelling microorganisms that play important roles in biodegradation, nutrient recycling, plant protection, and ecological balance. This study sought to understand how ZnO NPs affect *Bacillus subtilis*, a plant-beneficial bacterium ubiquitously found in soil. The impact of ZnO NPs on *B*. *subtilis* growth, FtsZ ring formation, cytosolic protein activity, and biofilm formation were assessed, and our results show that *B*. *subtilis* growth is inhibited by high concentrations of ZnO NPs (≥ 50 ppm), with cells exhibiting a prolonged lag phase and delayed medial FtsZ ring formation. RedoxSensor and P*_hag_*-GFP fluorescence data further show that at ZnO-NP concentrations above 50 ppm, *B*. *subtilis* reductase activity, membrane stability, and protein expression all decrease. SDS-PAGE Stains-All staining results and FT-IR data further demonstrate that ZnO NPs negatively affect exopolysaccharide production. Moreover, it was found that *B*. *subtilis* biofilm surface structures became smooth under ZnO-NP concentrations of only 5–10 ppm, with concentrations ≤ 25 ppm significantly reducing biofilm formation activity. XANES and EXAFS spectra analysis further confirmed the presence of ZnO in co-cultured *B*. *subtilis* cells, which suggests penetration of cell membranes by either ZnO NPs or toxic Zn^+^ ions from ionized ZnO NPs, the latter of which may be deionized to ZnO within bacterial cells. Together, these results demonstrate that ZnO NPs can affect *B*. *subtilis* viability through the inhibition of cell growth, cytosolic protein expression, and biofilm formation, and suggest that future ZnO-NP waste management strategies would do well to mitigate the potential environmental impact engendered by the disposal of these nanoparticles.

## Introduction

Zinc oxide nanoparticles (ZnO NPs) are known to be effective against many types of bacteria and fungi, both under ambient illumination and in the absence of ultraviolet (UV) light [1–7]. Antifouling paints have increasingly replaced bulk ZnO with ZnO NPs, due to their superior antibacterial properties [8]. Furthermore, the high catalytic activity of ZnO NPs make the substance an important industrial additive for many products, including plastics, cement, glass, rubber, lubricants, and food [9, 10]; and their excellent UV absorption and reflectivity have also made them a common component in cosmetics and sunscreens. In 2010, 550 tons of ZnO NPs were produced, making it the third most commonly used photocatalytic and antimicrobial agent, surpassed only by SiO_2_ and TiO_2_ NPs [11].

Various morphologies of ZnO NPs have been studied in order to elucidate the mechanisms underlying their antimicrobial effects, and although the precise mechanism remains unclear, several theories have been proposed, including the generation of reactive oxygen species (ROS) [4] or the release of cell membrane-damaging Zn^2+^ ions [12]. ROS are produced by ZnO NPs under light irradiation at frequencies of 368 nm or above [4, 13, 14], and can induce a range of biological responses in bacterial cells [15–17]. Studies have also shown that ZnO-NP antibacterial activity against *Escherichia coli* and *Candida albicans* may be due to lethal hydroxyl radicals generated by interactions between ZnO NPs and water [18, 19]. The effect of ZnO-NP particle size on antimicrobial efficiency has also been investigated [4, 20–22], and previous research showed that ZnO NPs less than 100 nm in size have more pronounced growth inhibitory effects than particles exceeding 1 μm [4]. Interestingly, gram-positive bacteria, such as *Staphylococcus aureus*, are more sensitive to ZnO NPs than gram-negative bacteria such as *Escherichia coli* [3, 22].

Although ZnO NPs may play a beneficial role when deployed against pathogenic microorganisms, they can adversely affect environmental bacteria, and are fully capable of altering the ecological balance in soil environments. Considering that bacteria are the primary decomposers in soil, environmental conditions that limit bacterial survival will have a negative impact on other organisms as well. *B*. *subtilis* is naturally found in the rhizosphere of grapevines and cereals [23, 24]. Moreover, *B*. *subtilis* has long been used as a biological control agent against different plant bacterial diseases [25, 26, 27]. *B*. *subtilis* can colonize the surfaces of plant roots, produce different types of lipopeptides against fungi, and activate the plant immune system against pathogens [28, 29]. Agricultural plant productivity is partly dependent upon such beneficial soil microbe activity, and growth disruptions in plant-beneficial bacteria could affect soil viability and interfere with plant growth. This study therefore sought to examine the effects of ZnO NPs on *B*. *subtilis*, a plant-beneficial bacterium ubiquitously present in soil. *B*. *subtilis* forms biofilms and spores in the soil environment, and is commonly used as a model organism to investigate the effects of ZnO NPs on microbial growth and protein activity [14, 20, 30, 31]. Several reports showed that *B*. *subtilis* cells failed to grow at ZnO-NP concentrations exceeding 200 ppm [20, 32]. At a lower concentration of 20 ppm, *B*. *subtilis* exhibits a prolonged lag phase. It has also been suggested that ZnO NPs may inhibit the activities of various enzymes, such as amylase and urease, although the related mechanisms are as yet unknown [32]. This study investigated the effects of ZnO NPs on the growth, protein expression, cell division, and biofilm formation of *B*. *subtilis*, and sought to elucidate the mechanisms underlying ZnO-NP impairment of bacterial viability.

## Materials and Methods

### ZnO-NP synthesis

Zinc acetate [Zn(CH_3_COO)_2_·2H_2_O, 32.85 g] was dissolved in 100 ml of distilled water with continuous stirring, until a homogeneous solution was obtained. This Zn-precursor solution (1.5 M) was then adjusted to a pH value of 9 by adding KOH (0.5 M) [33, 34, 35], and pulse microwave-assisted (MA) synthesis was performed as follows: The pH-adjusted Zn-precursor solution was placed in the center of a household microwave oven (Tatung Co., 900 W, 2.45 GHz, Taiwan) in which a thermocouple had been installed to detect reaction temperatures, and subjected to microwave irradiation at 700 W at 60°C for 30 min. The resulting precipitate was washed with distilled water 2–3 times, dried at 70°C for 4 h, crushed using a mortar and pestle, and calcinated in air at 500°C for 1 h.

### Characterization of ZnO NPs using X-ray diffraction (XRD) analysis and field emission scanning electron microscopy (FE-SEM)

Prepared samples were characterized in terms of structure, morphology, and elemental composition, using XRD analysis and FE-SEM. XRD was performed at a scanning range of 20–70° (2θ) on a Rigaku RU-H3R diffractometer, using Cooper Kα radiation with a wavelength of 1.5405 Å. FE-SEM was conducted with a JEOL JSM-6701F field emission scanning electron microscope.

### Fourier transform infrared (FT-IR) spectroscopy

FT-IR spectra of exopolysaccharide samples were obtained using a Bruker Tensor 27 FT-IR spectrometer with 32 scans in a frequency range of 4,000 to 600 cm^-1^.

### XANES and EXAFS spectroscopy

Overnight cultures of *B*. *subtilis* were treated with 100 ppm of ZnO NPs for 3 h, and cells were then pelleted by centrifugation. The cell pellets were washed three times with cold water and frozen at -80°C overnight. For freeze-drying, cells were desiccated under vacuum (50 mtorr) in a freeze-drier (Martin Christ, Germany) for 30 h. XANES and EXAFS spectra for the samples were directly collected at the Wiggler beam line 01C1 in the National Synchrotron Radiation Research Center (NSRRC) in Hsinchu, Taiwan. The electron storage ring was operated at an energy level of 1.5 GeV and a current of 100–200 mA. An Si(1 1 1) DCM was used to provide highly monochromatized photon beams with 6–33 keV (BL01C1) of energy and resolving power (E/△E) of up to 7,000. For ZnO (9,659 eV) K-edge experiments at room temperature, data were collected in fluorescence or transmission mode with a Lytle ionization detector. The photon energy was calibrated by characteristic pre-edge peaks in the absorption spectra of zinc standards. Local structural parameters, such as the bond length (R), coordination number (CN), and Debye-Waller factor (σ) for different coordination shells surrounding the absorbing atoms, were obtained through non-linear least-square fitting methods. The raw absorption data in the 50–200 eV region below the edge position was also fit to a straight line, using least-square algorithms. XANES spectra were extended to energy levels at the order of 50 eV above the edge. The EXAFS data were analyzed using the UWXAFS 3.0 program and FEFF 8.2 codes.

### *B*. *subtilis* strains and growth conditions

A *B*. *subtilis* wild type strain, 3610 [36], and its mutants, *sinR* [37], *epsA-O* [37], *tasA* [38], *srfA* [36], and *sinRepsA-O* (S1 Table) were maintained at 37°C in Luria-Bertani (LB; 10 g tryptone, 5 g yeast extract, and 5 g NaCl per liter) broth, or on LB plates containing 1.5% Bacto agar. ZnO NPs were added at the following concentrations where appropriate: 0, 5, 10, 25, 50, 100, and 200 ppm. For growth assays in a defined minimal medium for *B*. *subtilis*, wild-type 3610 was grown at 37°C in a minimal medium containing 7.0 g K_2_HPO_4_, 3.0 g KH_2_PO_4_, 0.1 g MgSO_4_·7H_2_O, 0.1 g (NH_4_)_2_SO_4_, 0.01 g CaCl_2_, 0.001 g FeSO_4_, 0.1 g NaCl, 1.0 g glucose, and 125 mg yeast extract per liter [39]. ZnO NPs were added at the following concentrations where appropriate: 0, 5, 10, 25, 50, and 100 ppm. For growth assays in biofilm medium, *B*. *subtilis* wild-type 3610 or mutant *sinR* were grown at 37°C in 1X SGG medium. ZnO NPs were added at the following concentrations where appropriate: 0, 5, 10, 25, 50, and 100 ppm.

### Time-dependent growth inhibition assay

To examine bacterial growth, overnight cultures of approximately 1 × 10^9^ CFU/ml were diluted 100-fold into 50 ml of LB broth in 250 ml flasks. ZnO NPs were added to the respective flasks at final concentrations of 0, 5, 10, 25, 50, and 100 ppm. Cultures were then grown for up to 30 h at 200 rpm, 37°C. Bacterial growth was measured by optical density at 600 nm (OD_600_). All experiments were performed in triplicate and averaged.

### Antibacterial activity of ZnO NPs against *B*. *subtilis*

Initial cultures (1 × 10^9^ CFU/ml) were prepared from 50-mL LB liquid cultures harvested at exponential growth. Bacterial cells were treated with ZnO NPs at increasing concentrations of 0–200 ppm for 9 h at 200 rpm, 37°C. Both treated and untreated cultures were then serially diluted and plated on LB agar plates at different time points. Plates were incubated overnight at 37°C and then subjected to a colony count. All experiments were performed in triplicate and averaged.

### Immunostaining of FtsZ rings in *B*. *subtilis* 3610

For FtsZ staining, 1 ml of bacterial culture was mixed with 10 ml of ice-cold 80% methanol for 1 h, and then fixed with 200 μl of 16% paraformaldehyde for 5 min at room temperature. After fixation, bacterial cells were centrifuged at 2,500 × *g* and suspended in 1 ml of 1X phosphate buffered saline (PBS) buffer. Coverslips were treated with 50 μl 0.1% poly-L-lysine solution for 10 min and air-dried. Fixed bacterial cells (200 μl) were placed on the treated coverslips, air-dried for 10 min at room temperature, and washed twice in 1X PBS buffer. The slides were then treated with freshly prepared lysozyme solution (2 mg/ml) in GTE buffer (50 mM glucose, 20 mM Tris-HCl, pH 7.5, 10 mM EDTA) for 5 min at room temperature, washed with PBS buffer, and subsequently incubated with BSA solution (PBS containing 2% BSA) overnight at 4°C. Slides were then incubated for 1 h at room temperature with anti-FtsZ antibody (1:200 dilution; Acris Antibodies, CA, USA), washed six times with PBS buffer, and incubated for 45 min with 200-fold diluted goat anti-rabbit IgG conjugated with Alexa Fluor 488 (Molecular Probes, Eugene, OR, USA). For membrane and nucleoid staining, slides were stained for 15 min with 1.5 μg/ml of FM1-43FX (Molecular Probes, Eugene, OR, USA) and 5 mg/ml of 4'-6-diamidino-2-phenylindole (DAPI; Molecular Probes, Eugene, OR, USA) in BSA solution. Slides were mounted with Citifluor AF1 mounting buffer (Agar Scientific, Essex, UK) and observed under a Leica TCS-SP2 laser-scanning confocal microscope at a magnification of 3,150X.

### Measurement of RedoxSensor activity

RedoxSensor activities of *B*. *subilis* 3610 were determined using a BacLight RedoxSensor Green Vitality Kit (Molecular Probes, Eugene, OR, USA). Overnight cultures of *B*. *subtilis* 3610 were treated with the indicated concentrations of ZnO NPs for 3 h at 37°C. Bacterial cells were then washed and diluted 10-fold in 1X PBS buffer, then mixed with 1 μl of RedoxSensor Green reagent and vortexed. To assess membrane integrity, 1 μl propidium iodide was added, and the mixture was incubated in the dark at room temperature for 5 min. Stained cells (10 μl) were spotted onto a clean slide and covered with a poly-L-lysine treated coverslip. Slides were observed under a Leica TCS-SP2 laser-scanning confocal microscope at a magnification of 630X.

### *P*_hag_-GFP expression at different concentrations of ZnO NPs

Plasmid pHag-gfp was constructed by inserting a DNA fragment containing the *gfp* sequence transcribed from the *hag* promoter into pHY300PLK (Takara, Shiga, Japan). *B*. *subtilis* 3610(P_*hag*_-GFP) was treated with ZnO NPs for 3 h at 37°C. Bacterial cultures (3 ml) were centrifuged and washed with 300 μl 1X T-Base buffer (15 mM (NH_4_)_2_SO_4_, 80 mM K_2_HPO_4_, 44 mM KH_2_PO_4_, 3.4 mM sodium citrate, and 3 mM MgSO_4_), and bacterial cells were resuspended in 50 μl 1X T-Base buffer containing 10 μg/ml of FM1-43FX and 5 mg/ml of DAPI. The mixture was incubated in the dark at room temperature for 15 min, and 4 μl of stained cells were spotted onto a clean slide and covered with a poly-L-lysine treated coverslip. Slides were observed under a Leica TCS-SP2 laser-scanning confocal microscope at a magnification of 3,150X.

### Flow cytometry analysis of *P*_hag_-GFP expression and RedoxSensor activity

For the *P*_hag_-GFP expression assay, bacterial cultures were treated with indicated concentrations of ZnO NPs for 3 h at 37°C, after which 3 mL of culture was centrifuged and washed with 300 μl 1X T-Base buffer. Bacterial cells were resuspended in 1 mL of 1X T-Base buffer, and flow cytometry was directly performed on a FACS Calibur flow cytometer (BD Biosciences, San Jose, CA, USA). Fluorescence filters and detectors were all standardized with green fluorescence collected in the FL1 channel (530 ± 15 nm). All parameters were collected as logarithmic signals.

For the RedoxSensor activity assay, bacterial cells were treated with the indicated concentrations of ZnO NPs, washed and diluted 10-fold in 1X PBS buffer, then mixed with 1 μl of a 1:10 dilution of RedoxSensor Green reagent (Molecular Probes, Eugene, OR, USA) and vortexed. To assess membrane integrity, 1 μl of a 1:10 dilution of propidium iodide (PI) was added, and the mixture was incubated in the dark at room temperature for 5 min. Samples (1 mL) were assayed by flow cytometry using a FACSCalibur flow cytometer (BD Biosciences, San Jose, CA, USA). Fluorescence filters and detectors were all standardized with green fluorescence collected in the FL1 channel (530 ±15 nm) and red fluorescence collected in the FL3 channel (> 650 nm). All parameters were collected as logarithmic signals. Data were analyzed using CellQuest Pro software. In density plots of light scatter properties, bacterial cells were gated from irrelevant counts for fluorescence analyses. Flow cytometry was calibrated using BD Calibrite beads (BD Biosciences, San Jose, CA, USA). Data are representative of results derived from two separate experiments.

### Biofilm assay

For pellicle formation experiments, 10 ml of 1X SGG broth (16 g/liter of nutrient broth (Difco/Becton Dickinson, MD, USA), 2 g/liter KCl, 0.5 g/liter MgSO_4_·7H_2_O, 1 mM Ca(NO_3_)_2_, 0.1 mM MnCl_2_·4H_2_O, 1 μM FeSO_4_, 0.1% glucose, and 1% glycerol) in 6-well microtiter plates was inoculated with 10 μl overnight culture grown at room temperature in LB medium. The culture was incubated at 25°C for 2 days [40, 41]. ZnO NPs were added at the following concentrations where appropriate: 0, 5, 10, 25, 50, 100, and 200 ppm.

### Extracellular polymeric substance (EPS) extraction

To assess the formation of exopolysaccharides, precipitation and staining of polymers present in culture supernatant were performed as described by Guttenplan et al [42]. Briefly, 1 ml of supernatant from overnight culture was treated with DNase and RNase to a final concentration of 67 μg/ml DNase I (Roche Diagnostics, Indianapolis, IN, USA) and 330 μg/ml Ribonuclease A (Sigma-Aldrich, St. Louis, MO, USA) for 30 minutes at 37°C, and subsequently treated with proteinase K to a final concentration of 400 μg/ml (Fisher Scientific, Rockford, IL, USA) for 1 h at 55°C. For staining samples using Stains-All (Sigma-Aldrich, St. Louis, MO, USA), the precipitate was spun down at 15,300 × g for 3 minutes. The supernatant was discarded, and the residual ethanol was allowed to evaporate. Each sample was mixed with 100 μl of 1X SDS sample buffer, and 20 μl was subsequently loaded onto a 12% SDS polyacrylamide gel. SDS-PAGE was performed for 30 minutes at 200 V. The stacking and resolving gel were fixed for 24 hours (25% isopropanol, 3% acetic acid), and stained overnight with 100 ml of Stains-All Reactive Solution (5 ml of 1 mg/ml Stains-All in formamide, 95 ml of Stains-All Base Solution (16.6% isopropanol, 5.5% formamide, and 0.5% 3.0 M Tris-HCl at pH 8.8), and 50 μl of 2-mercaptoethanol). For FT-IR analysis, overnight culture (500 μl) was subcultured to 50 ml of 1X SGG broth in 250 ml flasks, and incubated at 37°C for 24 h at 200 rpm. After culturing, cells were pelleted by centrifugation. The supernatant was placed on ice until chilled, and then mixed with ice-cold 75% ethanol overnight at 4°C. The precipitate was spun down at 8,440 × *g* for 10 min, after which the supernatant was discarded and the residual ethanol allowed to evaporate [42].

### SPP1 phage transduction

Serial dilutions of SPP1 phage stock were added to 0.2 ml of fresh dense culture grown in TY broth (LB broth supplemented with 10 mM MgSO_4_ and 100 μM MnSO_4_), and mixtures were statically incubated for 20 min at 37°C. Three ml of TYSA (molten TY supplemented with 0.5% agar) was added, poured atop fresh TY agar plates, and incubated at 37°C overnight. Top agar from the plate, containing near-confluent plaques, was scraped into a 50-ml conical tube, vortexed, and then centrifuged at 5,000 × *g* for 10 min. The supernatant was treated with 25 μg/ml DNase I before being passed through a 0.3-μm syringe filter and stored at 4°C. Recipient cells were grown to stationary phase in 2 ml TY broth at 37°C. The culture (0.9 ml) was mixed with 5 μl of SPP1 donor phage stock, and 9 ml TY broth was subsequently added to the mixture and allowed to stand at 37°C for 30 min. The transduction mixture was then centrifuged at 5,000 × *g* for 10 min, following which the supernatant was discarded and the pellet resuspended in the remaining volume of liquid. The cell suspension (100 μl) was then plated on TY fortified with 1.5% agar containing the appropriate antibiotic and 10 mM of sodium citrate. *sinRepsA-O* construct was derived from the wild-type *B*. *subtilis* strain 3610, using SPP1-mediated generalized phage transduction [43].

## Results

### Synthesis and morphological analysis of ZnO NPs

**Fig 1:**
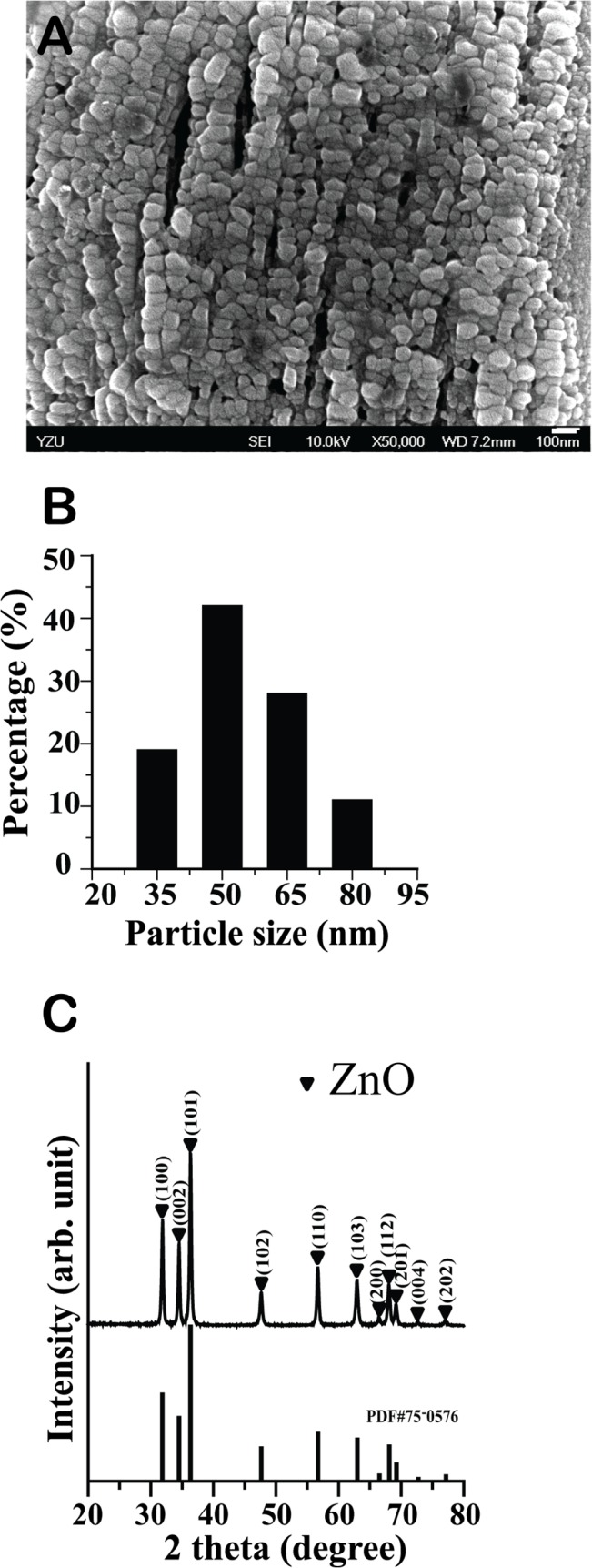
Synthesis and morphological analysis of ZnO NPs. **A**. Scanning electron microscope image of ZnO NPs used in this study; white bar: 100 nm. **B**. Size distribution of ZnO NPs. **C.** X-ray diffraction patterns of ZnO NPs synthesized by the sol-gel method.

ZnO NPs used in this study were synthesized using the sol-gel method [35] and examined by FE-SEM. FE-SEM images revealed that the average particle size of ZnO NPs was 50 nm (Fig 1A and 1B), and XRD results (Fig 1C) showed that the synthesized ZnO-NP powder had a pure wurtzite structure as indicated [3, 44]. ZnO NPs were then aggregated into a large population of hollow ZnO microspheres of approximately 4–5 μm in diameter (data not shown).

### ZnO NPs slow *B*. *subtilis* growth and delay FtsZ ring formation

**Fig 2:**
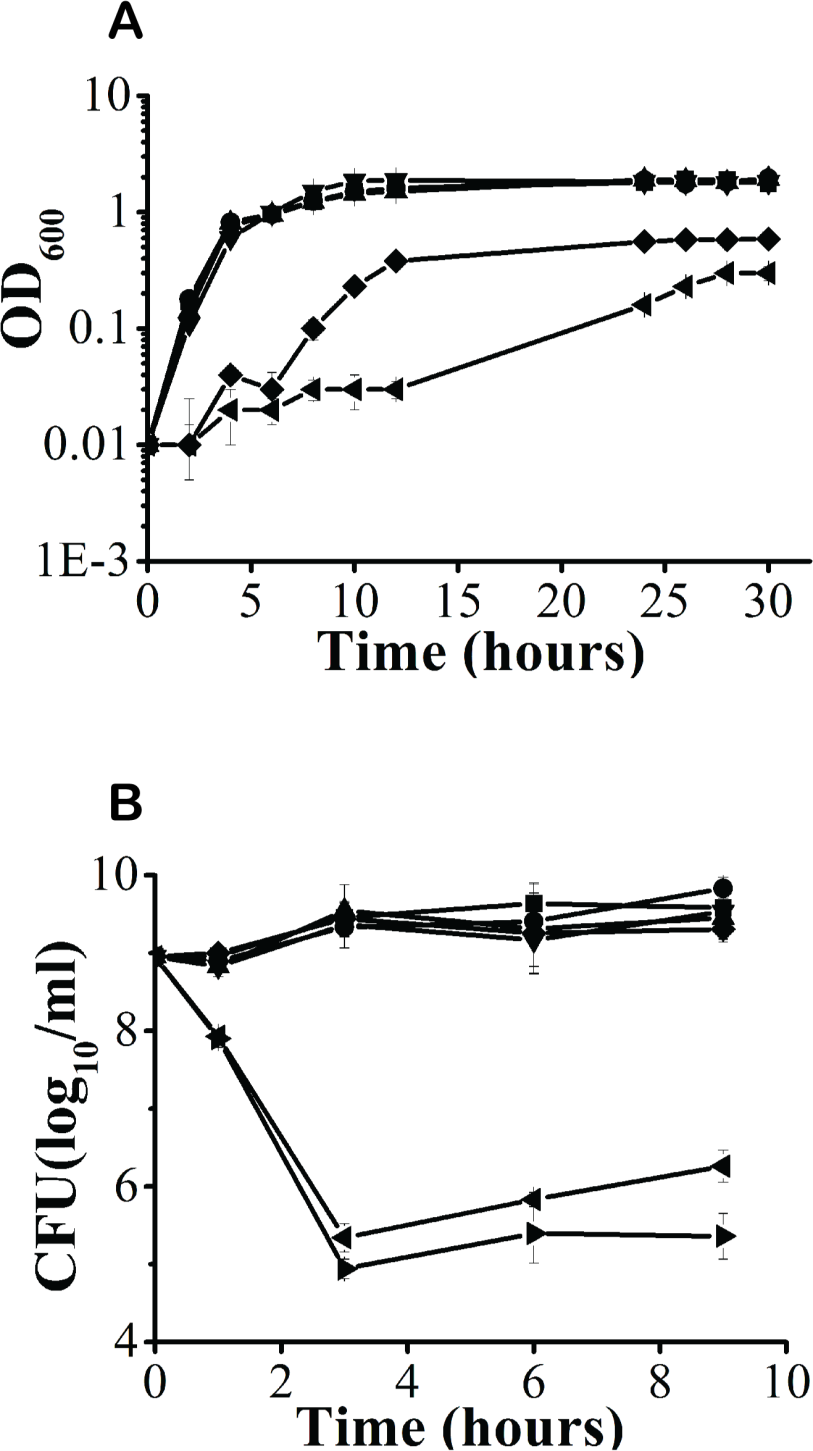
Effects of various ZnO-NP concentrations on the growth of *Bacillus subtilis*. **A.** Growth analysis curves were measured by monitoring the optical density (OD) at 600 nm. **B.** Antibacterial activity of ZnO NPs on *B*. *subtilis* cells; ZnO-NP concentrations are shown as -￭-: 0 ppm, -●-:5 ppm -▲-: 10 ppm, -▼-: 25 ppm, -◆-: 50 ppm, -◀-: 100 ppm, and -▶-: 200 ppm.

We treated *B*. *subtilis* 3610 cultures with 0–100 ppm of ZnO NPs, and evaluated bacterial growth over a period of 30 h in a rich LB medium. We found that 50 ppm of ZnO-NPs impaired bacterial growth for 6 h (Fig 2A). After 6 h, bacterial growth remained at reduced rates compared to controls, reaching an OD_600_ of approximately 0.58 at 30 h. At the highest ZnO-NP concentrations (100 ppm), limited cellular growth was observed in the first 12 h; however, growth rates accelerated somewhat after 24 h, though still remaining less than that of controls, with cells reaching an OD_600_ of approximately 0.3 at 30 h (Fig 2A). To mimic growth conditions in nature, we treated *B*. *subtilis* 3610 cultures with 0–100 ppm of ZnO NPs, and evaluated bacterial growth over a period of 30 h in a low-nutrient minimal medium. In cultures grown with 10–50 ppm concentrations of ZnO NPs, although cells initially grew more slowly than controls cultivated without ZnO NPs, catch-up growth was observed after 4 h, and the OD_600_ was comparable to controls by 12 h (S1 Fig). However, when cultivated with 100 ppm of ZnO NPs, almost no bacterial growth was observed during the first 10 h, with only slight growth seen after 12 h; the OD600 at 30 h was 0.1 (S1 Fig). This suggests that at concentrations up to 100 ppm, ZnO NPs can exert a slowing effect on *B*. *subtilis* growth.

To ascertain that the reduced growth rates observed in *B*. *subtilis* were not caused by interactions between ZnO NPs and the LB medium, we pretreated LB medium with varying concentrations of ZnO NPs for 30 hrs with moderate shaking. Cultures of *B*. *subtilis* in this pretreated medium displayed similar growth patterns to cultures in non-pretreated medium (S2 Fig), indicating that ZnO NPs and LB medium likely do not interact to mutually affect their inherent properties.

Lethal effects were observed in *B*. *subtilis* cells at ZnO-NP concentrations of 100 ppm or greater. We added ZnO NPs to 10^9^ CFU/ml of freshly grown culture, and incubated the mixture for 9 h. We found that treatment with 100 or 200 ppm of ZnO NPs resulted in a 4-log10 CFU/ml reduction in population size (Fig 2B). These results indicate that *B*. *subtilis* growth and entry to the exponential phase are delayed after treatment with 10–100 ppm of ZnO NPs, with significant lethality observed following treatment with ZnO-NP concentrations of 100 and 200 ppm.

**Fig 3:**
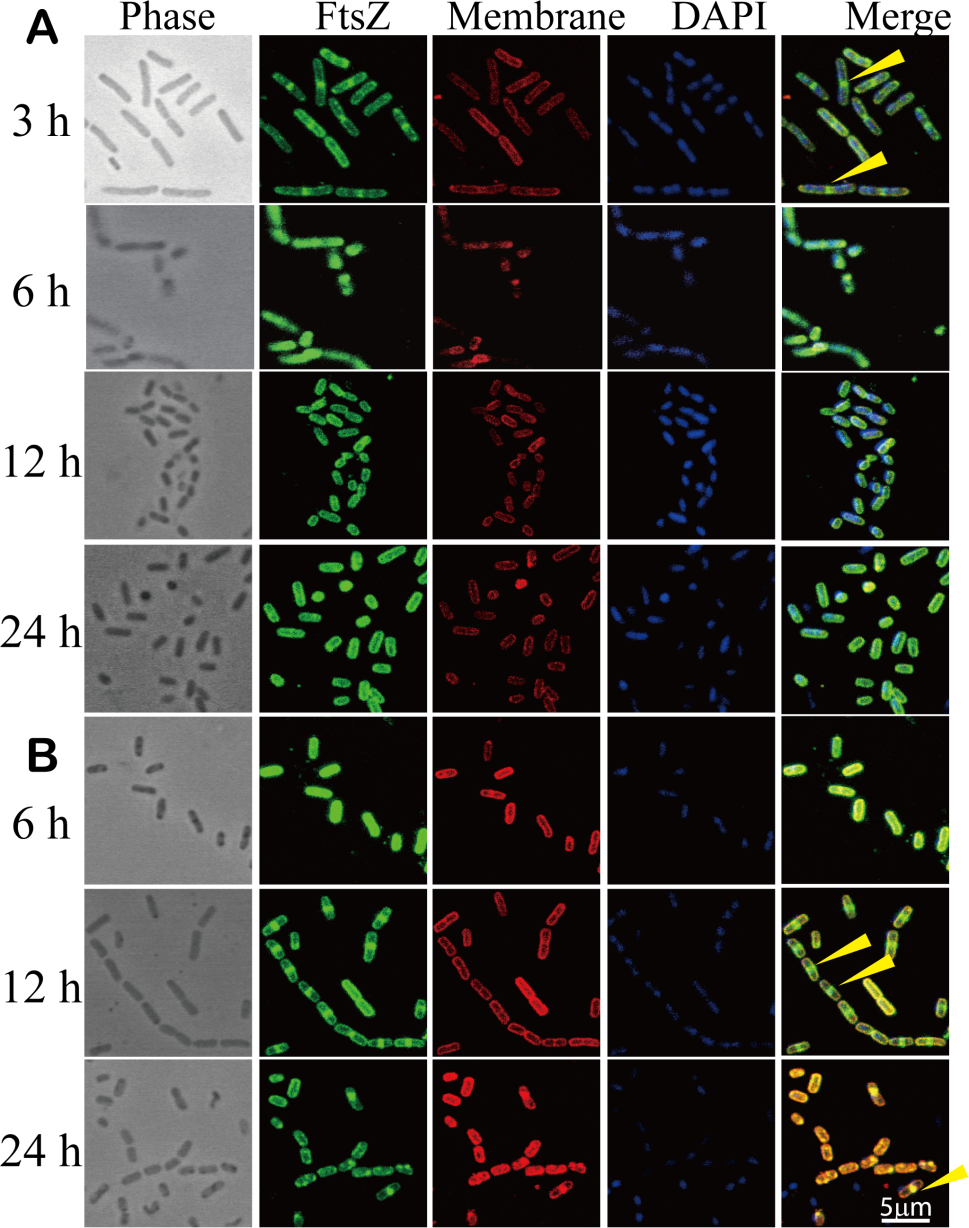
Localization of FtsZ in wild-type cells grown under concentrations of 0, 50, and 100 ppm ZnO NPs in LB at 37°C. FtsZ was stained green; cell membranes were stained red; and DNA was stained blue. The closed yellow arrows indicate medial FtsZ rings.

We sought to understand whether ZnO NPs inhibited growth in *B*. *subtilis* by affecting cell division. *B*. *subtilis* was stained with FtsZ antibodies (green) to determine the timing and position of FtsZ ring formation; membranes and nucleoids were stained with FM1-43X (red) and DAPI (blue), respectively. *B*. *subtilis* cells were grown under the same conditions as shown in Fig 2A. We counted 300 cells at five different sites in separate experiments to derive the percentage of FtsZ rings. In bacterial cells cultured without ZnO NPs, during the mid-exponential phase, which occurs about 3 h after inoculation, 35% were observed to have FtsZ rings (Fig 3A); however, FtsZ rings were undetectable during the stationary phase (12 h and 24 h post-inoculation), when cell division ceases. In contrast, cultures treated with 50 ppm of ZnO NPs did not form FtsZ rings at 6 h post-inoculation (Fig 3B). FtsZ rings (observed in 63% of cells) began to appear at 12 h post-inoculation, but at 24 h post-inoculation, only a very low percentage of cells had FtsZ rings (1%). Cells treated with 100 ppm of ZnO NPs barely grew at 6 h post-inoculation (Fig 2A) and did not form FtsZ rings (S5 Fig). FtsZ rings began forming only at 24 h post-inoculation (seen in 26% of cells), but slow growth continued to be demonstrated even at mid-log phase (S5 Fig). These results suggest that ZnO-NPs slow cell growth and delay FtsZ ring formation, which may subsequently lead to the prolonged lag phase (lasting up to 12 h post-inoculation) observed in bacterial cells cultured with 50 or 100 ppm of ZnO NPs.

### ZnO NPs reduced RedoxSensor activity and P_*hag*_-GFP expression

**Fig 4:**
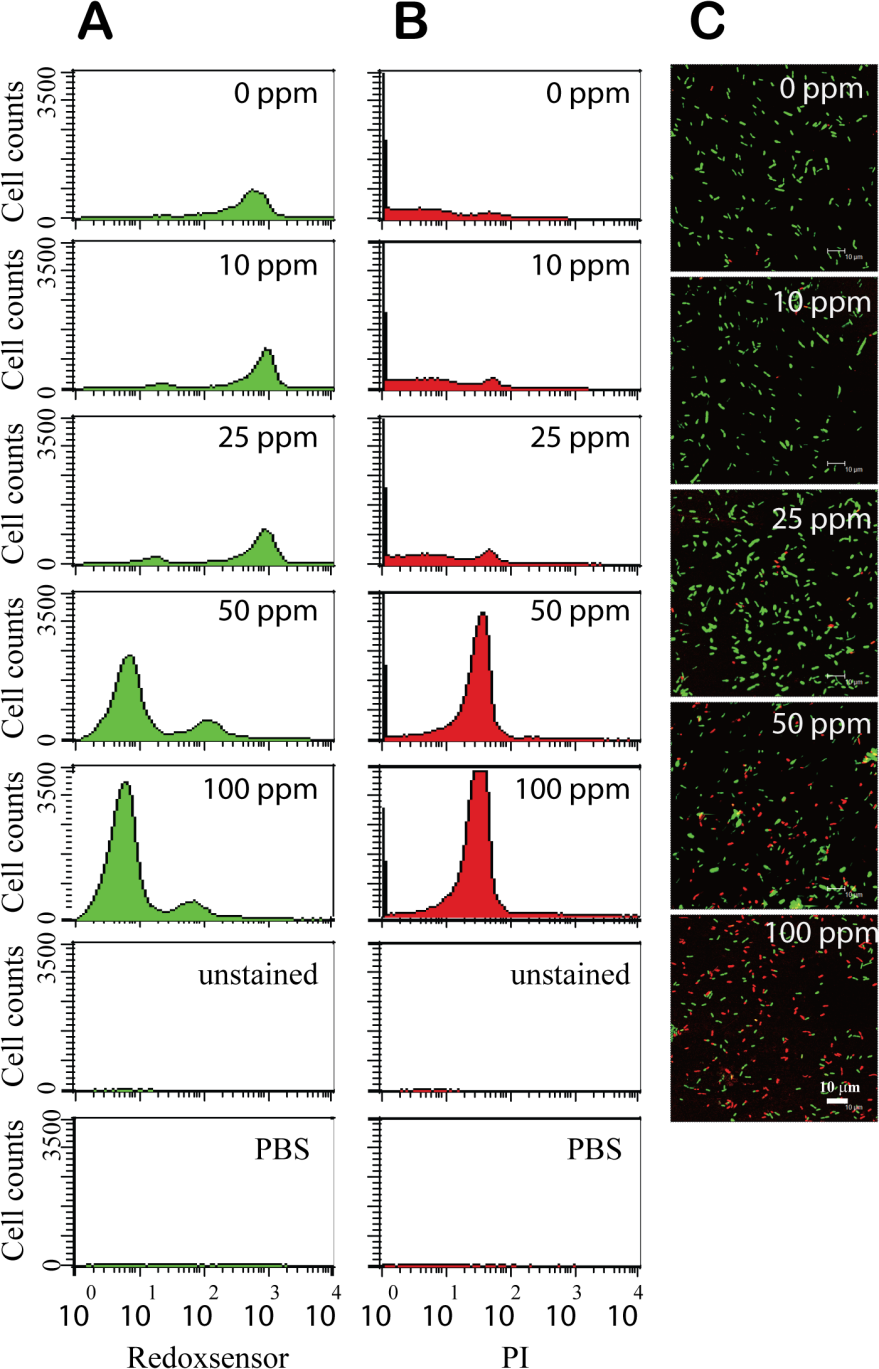
Flow cytometry and fluorescent micrograph analysis of RedoxSensor activity in *B*. *subtilis*. Wild-type bacteria were grown for 3 hrs at ZnO-NP concentrations of 0 ppm, 10 ppm, 25 ppm, 50 ppm, 100 ppm. Unstained samples and PBS buffer alone were used as controls. The X axis indicates RedoxSensor or PI fluorescence intensity (arbitrary units: au), and the Y axis indicates cell counts. **A.** Flow cytometry analysis of Redoxsensor activity, and **B.** Flow cytometry analysis of PI activity. **C.** Fluorescent micrographs of *B*. *subtilis* cells indicate RedoxSensor activity or PI fluorescence after incubation with different concentrations of ZnO-NPs for 3 h. Redoxsensor activity presents a false green color. PI presents a false red color. Scale bar: 10 μm.

To determine whether reductase activity was affected by ZnO NPs, *B*. *subtilis* cells were grown to early stationary phase, at approximately 10^9^ CFU/ml, and varying concentrations of ZnO NPs were then added. After 3 h of treatment, bacterial cells were washed, and the reductase activity and membrane integrity of approximately 10^9^ *B*. *subtilis* CFUs were respectively assessed with RedoxSensorGreen (green) and PI (red) staining. From the microscopic data shown in Fig 4C, significant differences in RedoxSensor Green (green) activity were seen in cultures cultivated with 0–100 ppm of ZnO NPs. Moreover, cells treated with ZnO-NPs at concentrations ≥ 50 ppm for 3 h exhibited increased PI fluorescence (red, Fig 4C). We further quantified RedoxSensor activity using flow cytometry. Bacterial cells were grown to early stationary phase at approximately 10^9^ CFU/ml, treated with varying concentrations of ZnO NPs for 3 h, stained, and then subjected to flow cytometry. Results confirmed that RedoxSensor activity decreased following treatment with 50 or 100 ppm of ZnO-NPs (Fig 4A). RedoxSensor levels (intensity of X geometric mean at LR region; % of gated cells) for cells treated with varying concentrations of ZnO NPs were as follows (Fig 4A): 0 ppm: (496.98; 81.9%); 5 ppm: (512.63; 75.27%); 10 ppm: (573.83; 79.21%); 25 ppm: (543.88; 80.28%); 50 ppm: (73.41; 17.64%); and 100 ppm: (71.38; 10.57%). PI levels (intensity of Y geometric mean at LL region; % of gated cells) for cells treated with varying concentrations of ZnO NPs were as follows (Fig 4B): 0 ppm: (34.63; 15.16%); 5 ppm: (38.84; 20.58%); 10 ppm: (37.05; 17.54%); 25 ppm: (31.57; 17.22%); 50 ppm: (20.53; 79.57%); and 100 ppm: (25.68; 86.24%). Specifically, at 50 ppm and 100 ppm, RedoxSensor content dramatically decreased and PI intensity slightly decreased, but the percentage of gated cells increased significantly. The flow cytometry data is representative of two separate experiments. This suggests that high concentrations of ZnO NPs affect RedoxSensor activity and cell membrane permeability. As shown in Fig 2B, 100 and 200 ppm of ZnO-NPs exerted a lethal effect on bacterial cells. At 50 ppm or less, although significant lethality was not observed (Fig 2B), ZnO-NPs significantly decreased reductase activity, and probably induced membrane perforation (Fig 4A and 4B).

**Fig 5:**
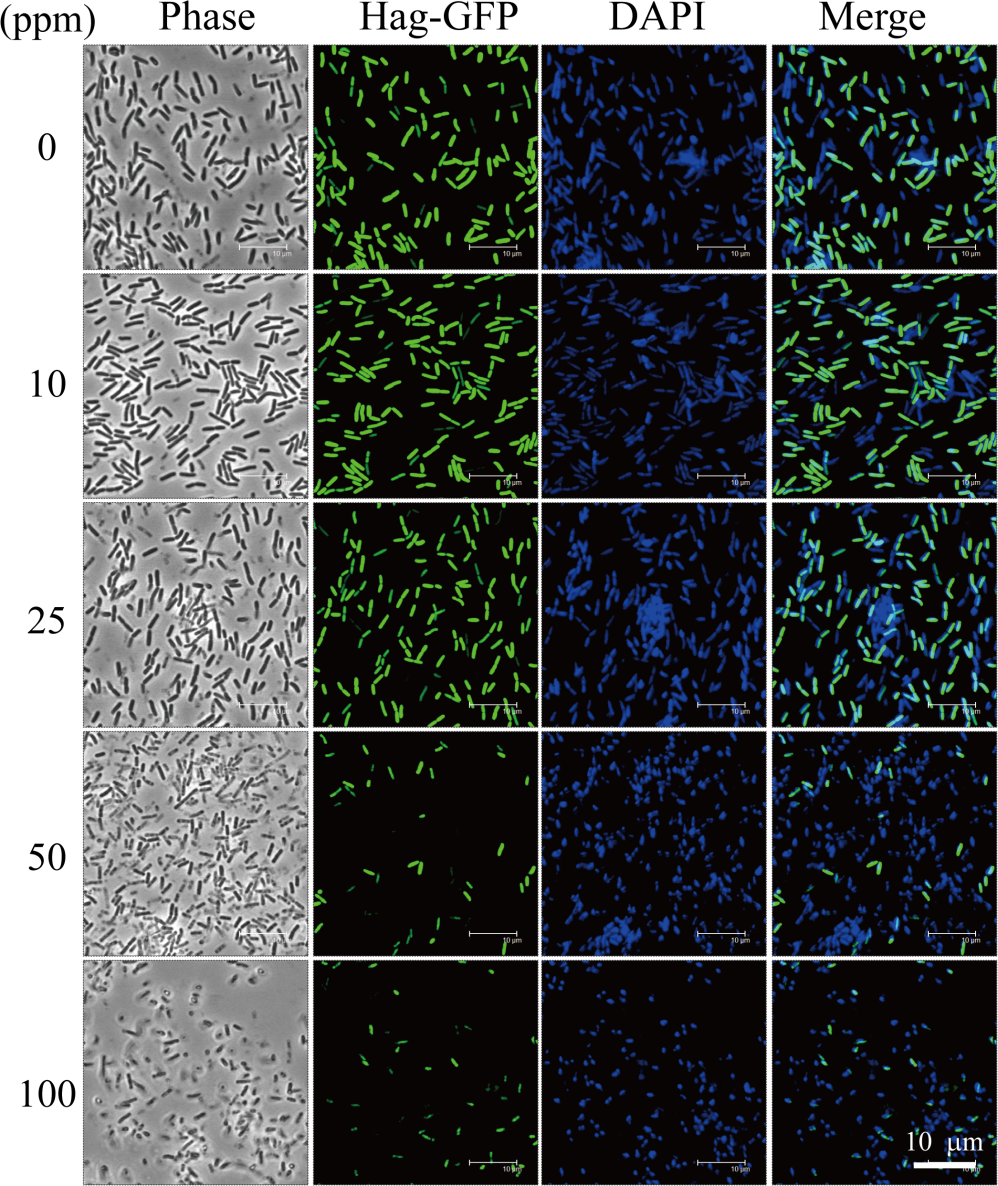
*P*_*hag*_ *-GFP* at varying concentrations of ZnO NPs. Fluorescent micrographs of *B*. *subtilis* cells show the expression of *P*_***hag***_ *-GFP* after cultivation with ZnO-NP concentrations of 0, 5, 10, 25, 50, and 100 ppm for 3 h. GFP reporter expression presents a false green color. DAPI presents a false blue color. Scale bar: 10 μm.

We also sought to ascertain the effect of ZnO NPs on protein expression. *B*. *subtilis* 3610 (P_*hag*_-GFP) was grown until early stationary phase. Cultures were then incubated with varying concentrations of ZnO NPs for 3 h. At lower concentrations of ZnO NPs (5, 10, and 25 ppm), no significant difference in GFP expression, compared to controls, was observed (Fig 5). However, at ZnO-NP concentrations of 50 and 100 ppm, a dramatic decrease in GFP expression was seen, and DAPI staining revealed that the integrity of chromosomal DNA was severely compromised (Fig 5). These results suggest that ZnO-NP concentrations of 50 ppm can exert a critical effect on P_*hag*_-GFP and cytosolic protein expression in *B*. *subtilis* cells.

**Fig 6:**
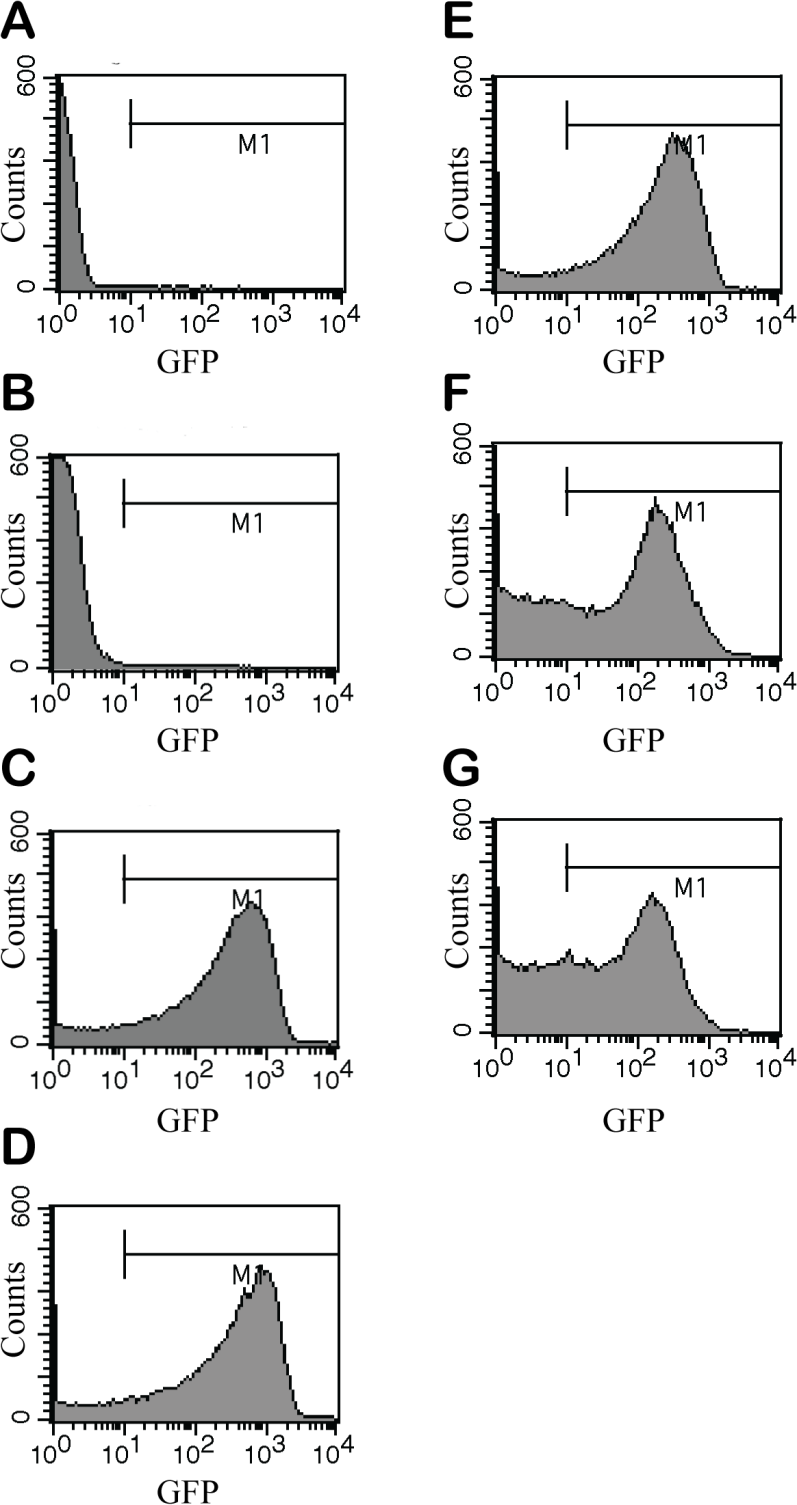
Flow cytometry analysis of *P*_*hag*_ *-GFP* at various concentrations of ZnO NPs. Wild-type bacteria were grown at different ZnO-NP concentrations for 3 h. **A**: PBS, **B**: unstained cells, **C**: 0 ppm, **D**: 10 ppm, **E**: 25 ppm, **F**: 50 ppm, and **G**: 100 ppm. The X axis indicates GFP fluorescence intensity (arbitrary units: au), and the Y axis indicates cell counts.

We further quantified P_*hag*_-GFP expression using flow cytometry. Bacterial cells were grown to early stationary phase at approximately 10^9^ CFU/ml, treated with varying concentrations of ZnO NPs for 3 h, stained, and subjected to flow cytometry. Results confirmed that levels of P_*hag*_-GFP expression were decreased after treatment with 50 and 100 ppm of ZnO NPs (Fig 6F and 6G). P_*hag*_-GFP levels (intensity of X geometric mean at LR region; % of gated cells) for cells treated with varying concentrations of ZnO NPs were as follows (Fig 6C, 6D, 6E, 6F and 6G): 0 ppm: (275.29; 83.04%); 10 ppm: (334.77; 84.27%); 25 ppm: (197.50; 79.07%); 50 ppm: (128.15; 57.62%); and 100 ppm: (89.69; 54.92%). Specifically, at 50 ppm and 100 ppm, P_*hag*_-GFP levels dramatically decreased, while the percentage of gated cells rose significantly. This suggests that high concentrations of ZnO NPs can affect P_*hag*_-GFP expression. The flow cytometry data is representative of two separate experiments.

### ZnO NPs prevent biofilm formation

**Fig 7:**
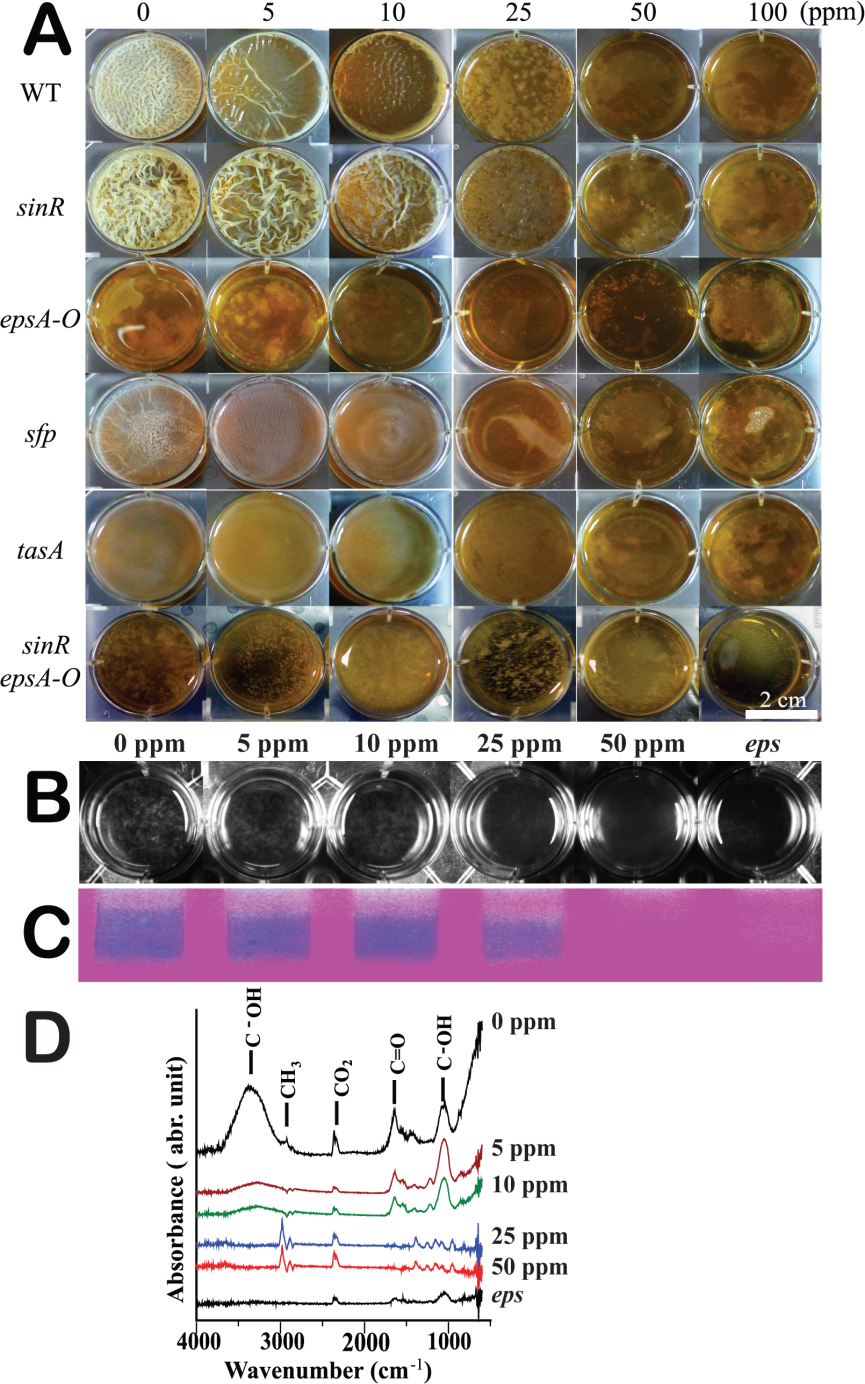
ZnO NPs affect biofilm formation. **A.** The pellicle column depicts microtiter wells (6-well plate) in which cells were grown in biofilm medium with various concentrations of ZnO NPs at 25°C for 3 days (scale bar: 2 cm). Bacterial wild-type (3610) and mutant strains are indicated as follows: *sinR* (DS92), *epsA-O* (DS696), *sfp* (DS3629), *tasA* (DS3630), and *sinR epsA-O* (HS222). **B.** Images of a 12-well microtiter dish containing ethanol-precipitated supernatant from the indicated strain, following treatment with different concentrations of ZnO NPs. **C**. The supernatants of the indicated strains were treated with proteinase K, DNase, and RNase, precipitated with ethanol, and resolved through SDS-PAGE on a 12% gel, after which staining with Stains-All was performed. **D.** FT-IR spectra analysis of EPS from *Bacillus* cells treated with ZnO NPs. Wild type bacteria were grown at ZnO NP concentrations of 0, 5, 10, 25, and 50 ppm, and untreated *eps* mutant cells were grown to serve as a negative control.

The ability to form biofilms is an important characteristic of many soil bacteria, including *B*. *subtilis*. After treating *B*. *subtilis* with 25, 50, or 100 ppm of ZnO NPs, wild-type *B*. *subtilis* cells were unable to form biofilms, although thin biofilms were observed following treatment with 5 or 10 ppm of ZnO-NPs (Fig 7A). The *sinR* mutant is a biofilm hyper-producer that secretes more EPS (exopolysaccharides) than wild-type strains. This mutant was shown to be resistant to ZnO NPs at concentrations of 5 and 10 ppm, forming biofilms thicker than those produced by its wild-type counterparts. Conversely, the *epsA-O* mutant is a biofilm-deficient strain that does not produce EPS, and this mutant did not form biofilms at any ZnO-NP concentrations. The *sfp* mutant (related to surfactin expression) as well as the *tasA* mutant (a biofilm anchor-deficient strain) both produced relatively thinner biofilms than wild-type cultures at ZnO-NP concentrations of 5 and 10 ppm. At concentrations exceeding 10 ppm, these strains were unable to form biofilms. We therefore concluded that wild-type bacterial strains are able to form thin biofilms under low ZnO-NP concentrations of 5 and 10 ppm, but biofilm formation is inhibited at concentrations exceeding 25 ppm.

In addition, we posited that the *sinR* mutant should be relatively impervious to ZnO NPs at higher concentrations, compared to wild-type strains and biofilm-deficient mutants. To determine whether elevated EPS production in *sinR* mutants increases resistance to ZnO NPs, we mutated the *epsA-O* genes in the *sinR* mutant, which resulted in deficient biofilm production upon exposure to ZnO NPs at any concentration (Fig 7A). These results support the hypothesis that the resistance of the *sinR* mutant to ZnO NPs can be attributed to increased EPS production. This resistance did not result from differences in cell growth, as a growth curve assay comparing wild-type cells and *sinR* mutants grown in biofilm medium with shaking was conducted, and no significant difference between the two strains was observed (S3 Fig and S4 Fig).

We further sought to assess whether ZnO NPs would affect EPS production. Ethanol precipitation was used to derive EPS from the culture supernatant of wild-type strains and *eps* mutants (Fig 7B). In supernatant from cultures treated with 25 or 50 ppm concentrations of ZnO NPs, no EPS precipitation was observed (Fig 7B). EPS production was also examined by SDS-PAGE (Fig 7C). Surprisingly, a major negative effect on the amount of EPS was observed when cells were grown at 25 or 50 ppm of ZnO NPs (Fig 7C). A slight decrease in EPS levels was observed when cells were grown at 5 or 10 ppm of ZnO NPs. The *epsA-O* mutant, which does not produce EPS, was used as a negative control. To exclude the possibility that decreases in EPS production were caused by growth inhibition at higher ZnO-NP concentrations, wild-type bacterial cells were grown in biofilm medium (SSG medium), and these presented no significant differences in growth behavior (S3 Fig) when exposed to ZnO NPs at concentrations of 5, 10, or 25 ppm. Only at ZnO-NP concentrations of 50 or 100 ppm were cells observed to grow more slowly than cultures grown at lower ZnO-NP concentrations (S3 Fig). From these results, it was concluded that since ZnO-NP concentrations starting from 10 ppm were shown to decrease EPS production, this decrease cannot be attributed to a reduction in *B*. *subtilis* growth rates, as no growth differences were observed in cultures grown at ZnO-NP concentrations of 0 to 25 ppm (S3 Fig).

The functional groups of EPS molecules were analyzed with FT-IR. The FT-IR spectrum displayed a broad-stretching intense peak at 3,414 cm^−1^, indicative of hydroxyl groups, in wild-type strains grown without ZnO NPs (Fig 7D). Two weak symmetrically-stretching peaks at 3,000 and 2,400 cm^−1^ respectively indicate the presence of methyl and carbon dioxide groups. The absorption peaks around 1,700 cm^−1^ further indicate the presence of a carbonyl group, and the absorption peak around 1,000–1,100 cm^−1^ is known to be characteristic of all sugar moieties and carboxyl groups [45, 46]. The observed carboxyl and hydroxyl groups are representative of the EPS molecule. In Fig 7D, a reduction in the 1,000–1,100 cm^−1^ peaks, representing the carbonyl and carboxyl groups, was observed in cultures grown under ZnO-NP concentrations of 25 and 50 ppm, but not for those cultured with 0, 5, or 10 ppm of ZnO NPs. FT-IR analysis of EPS production in *eps* mutants and wild-type bacteria cultured under ZnO-NP concentrations above 25 ppm did not present peaks at 1,000–1,100 cm^−1^ or 1,700 cm^−1^, indicating a lack of EPS production.

### XANES/EXAFS analysis of ZnO-NP fine structure in *B*. *subtilis* cells

**Fig 8:**
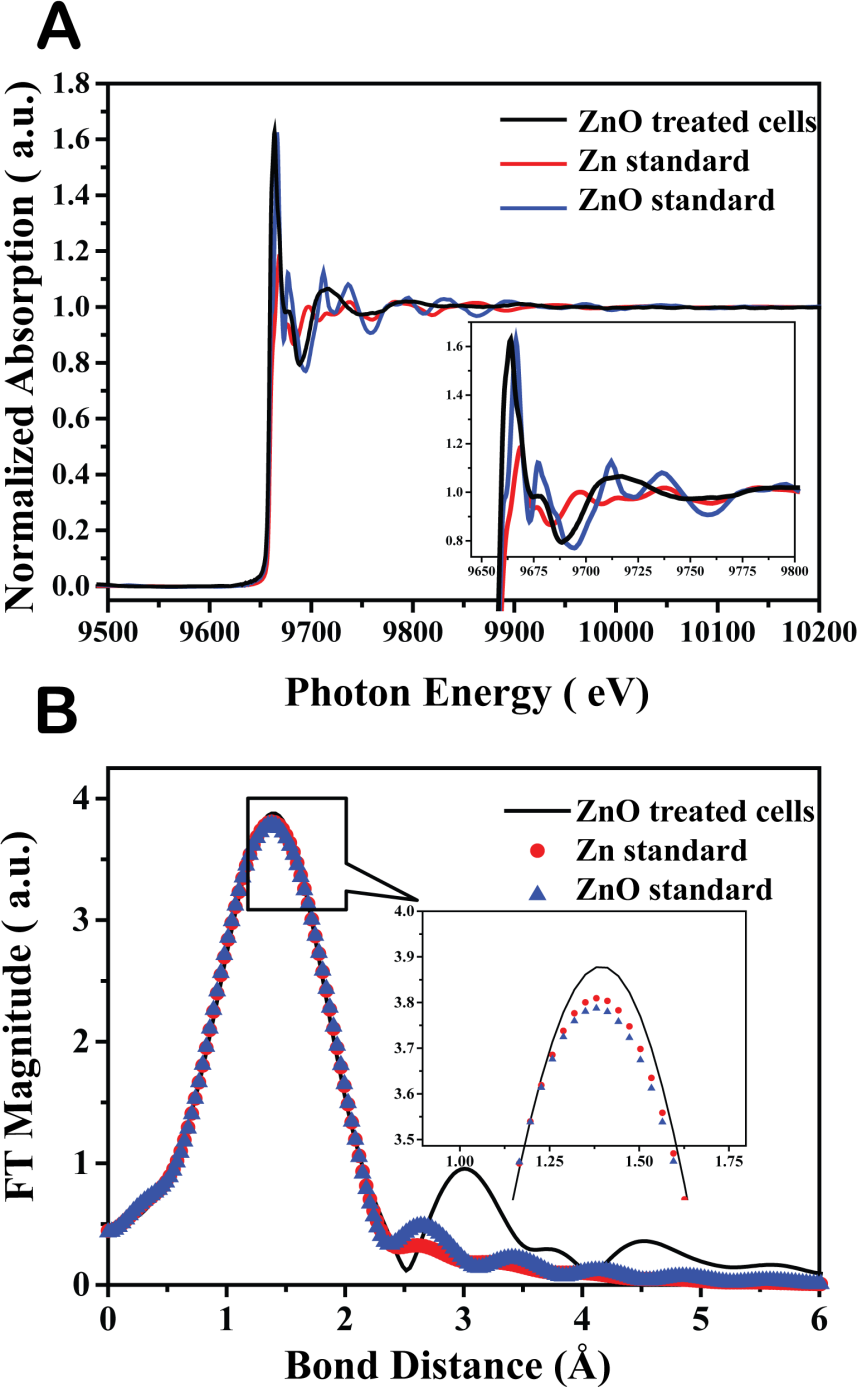
XANES and EXAFS spectra for *B*. *subtilis* cells treated with ZnO NPs. **A.** ZnO K-edge XANES spectra of silver standards and *B*. *subtilis* cells treated with 100 ppm of ZnO-NPs. **B.** ZnO K-edge EXAFS spectra of ZnO standards and *B*. *subtilis* cells treated with 100 ppm of ZnO-NPs. The best-fitting EXAFS spectra are indicated by the colored symbol lines. (Zn-Zn standard: red; ZnO standard: blue; *Bacillus subtilis* cells treated with 100 ppm of ZnO-NPs: black).

To investigate the oxidation state and fine structure of ZnO-NPs in *B*. *subtilis* cells, we conducted both XANES and EXAFS spectra analysis. The normalized zinc K-edge spectra of ZnO-NPs in treated *B*. *subtilis* cells and ZnO standards (Zn, ZnO) are presented in Fig 8A. There were several sharp absorption peaks of ZnO standards in the range between 9,650 and 9,775 eV. At 9,664 eV, the pattern of ZnO-NPs (50 ppm) in treated *B*. *subtilis* cells (black) fitted the ZnO standard (blue). The photon energy (1 eV) shift was likely due to changes in Zn-O bond length in the *B*. *subtilis* cells. These XANES results demonstrate that ZnO-NPs were truly present in *B*. *subtilis* cells.

The bond length (R), and coordination number (CN) of ZnO-NPs were investigated by EXAFS. The EXAFS spectra of ZnO-NPs (50 ppm) in treated *B*. *subtilis* cells and ZnO standards had a single peak between 0–2 Å, the position of the first shell in zinc compounds. The peaks between 0–6 Å represented the different coordination shells, including Zn-O (ZnO) and Zn-Zn (Zn), respectively shown in Fig 8B. The spectra of ZnO-NPs (50 ppm) in treated cells (black), along with ZnO (blue) and Zn (red), overlapped at 1.38 Å, which was suggestive of the coordination shell belonging to Zn-O or Zn-Zn. To obtain further fine structure parameters (bond length and coordination number), cells treated with 100 ppm of ZnO-NPs were analyzed by EXAFS, with the results shown in S2 Table.

The spectra of cells treated with 50 ppm of ZnO-NPs (black) overlapped with ZnO (blue) at 1.38 Å. Bond lengths and coordination number of cells treated with 50 ppm ZnO-NPs (black) were 2.05 and 3.9 Å, respectively. In order to confirm the coordination shell, the structural parameters after EXAFS model fitting were examined. In S2 Table, the Zn-Zn shell model was irrational because of its oversized coordination number (59.4). In contrast, the Zn-O shell model (coordination number 3.9) was more acceptable, and R-factor results further confirmed that the Zn-O shell model (0.002) was more precise than the Zn-Zn shell model (0.006). The coordination shell was revealed as belonging to Zn-O; specifically, Zn (II)-O. It was previously shown that ZnO NPs dissolved in LB medium may leach Zn^2+^ ions [47], and it has been proposed that these Zn^2+^ ions will attach to cell membranes and lead to membrane damage, an ROS response, and protein dysfunction [48]. The XANES and EXAFS results here confirm that ZnO is present in *B*. *subtilis* cells cultivated with ZnO NPs, suggesting that toxic Zn^2+^ ions from ionized ZnO NPs may have permeated cell membranes and were later deionized to ZnO, or that ZnO NPs may be directly capable of penetrating cell membranes in the original ZnO state.

## Discussion

This study investigated the effects of ZnO NPs against *B*. *subtilis* planktonic and biofilm cells, to elucidate the physiological consequences of exposure to ZnO NPs. We found that ZnO-NP concentrations exceeding 50 ppm slow bacterial growth and prolong the lag phase. These results are consistent with findings made by previous studies, which showed that *B*. *subtilis* growth has a longer lag phase when treated with ZnO-NP concentrations of 162.6 ppm (ZnO < 1 μm) or 20 ppm (ZnO < 35 nm) over a period of 12 h, as compared to untreated cells [32, 49]. Since smaller nanoparticles are more toxic to cells and can therefore inhibit cell growth at lower concentrations [12, 15], the lower inhibitory concentrations seen with ZnO NPs of 35 nm are to be expected, as this study used ZnO NPs of approximately 50 nm in size, which necessitated greater concentrations of ZnO NPs to limit growth. Moreover, Santimano et al. [32] demonstrated that ZnO-NP concentrations at 200 ppm are lethal to *B*. *subtilis*, and we also observed similar phenomena (Fig 2B).

Previous studies have shown that ZnO NPs dissolved in LB medium may leach Zn^2+^ ions [47], and it has been suggested that these Zn^2+^ ions can attach to cell membranes to induce membrane damage, an ROS response, and protein dysfunction [48]. Huang et al. [12] showed that ZnO NPs cause membrane damage and membrane disorganization in *Streptococcus agalactiae* and *Staphyloccocus aureus*. We also used *in vivo* PI staining to examine membrane integrity. At ZnO-NP concentrations exceeding 50 ppm, the number of cells with membrane damage increased (Fig 4B and 4C); but as PI assays are not capable of ascertaining bacterial mortality rates, we were thus unable to determine whether membrane degradation occurs as a result of ZnO NP-induced cell death, or whether ZnO NPs permeabilize membranes and subsequently undermine bacterial cell viability. As we have shown that ZnO NPs reduce cytosolic protein expression and reductase activity at concentrations of 50 ppm, it is possible that ZnO-NP treatment either damaged cellular membranes, resulting in leakage; or penetrated bacterial cell membranes to cause protein dysfunction and/or decreased protein expression. Interestingly, the number of bacterial cells did not dramatically decrease with the addition of 50 ppm of ZnO NPs (Fig 2B). The XANES/EXAFS data show that ZnO is present in bacterial cells cultivated with ZnO NPs. It is possible that Zn^+^ ions entered *B*. *subtilis* cells to induce an ROS response, resulting in decreased RedoxSensor activity, but were subsequently deionized to ZnO. This might explain the results observed in cultures cultivated with ≥ 50 ppm of ZnO NPs, where RedoxSensor activity and PI intensity both decreased, but the percentage of gated cells increased significantly.

Bacteria form biofilms on a variety of surfaces, and become more resistant to antibacterial agents than planktonic cells [50, 51, 52]. We compared the effects of ZnO NPs on wild type *B*. *subtilis* and *sinR* mutants. The *sinR* mutant proved highly resistant to ZnO NPs at concentrations ranging from 1–25 ppm; by contrast, biofilm-deficient strains, such as *eps*, *sfp*, and *tasA* mutants, were more susceptible to ZnO NPs. In addition, the *sinR* mutant produced more EPS molecules than wild-type strains, and wild-type bacteria were found to produce less EPS when grown at ZnO-NP concentrations exceeding 10 ppm. Therefore, we conclude that the production of EPS plays a role in resistance to ZnO NPs, as EPS may serve as a natural barrier that offers protection from the detrimental effects of ZnO NPs. It is worth noting that ZnO NPs decreased biofilm formation from concentrations as low as 25 ppm. This could have a negative effect on *B*. *subtilis* biofilm formation capabilities on plant root surfaces, and subsequently lead to decreased protection against plant pathogens. The exposure of soil to ZnO NPs might potentially alter rhizosphere ecosystems and increase the vulnerability of plants to disease, resulting in poor growth and decreased agricultural productivity.

Cells may cease growing and undergo a prolonged lag phase when cell division is disrupted. Therefore, we conducted immunostaining assays to observe the effect of ZnO NPs on FtsZ ring formation. In the absence of ZnO NPs, cells formed FtsZ rings at 3 h post-inoculation, during the mid-exponential phase. However, with the addition of 50 or 100 ppm of ZnO NPs, bacterial cells respectively formed FtsZ rings at 12 h and 24 h post-inoculation. This is the first study to demonstrate that ZnO NPs can retard cell division. The mechanism of why ZnO NPs affect FtsZ formation remains unclear; but taken in perspective, if the growth of soil microorganisms were to be affected by exposure to ZnO NPs, resistant microorganisms could rapidly become dominant, thereby altering soil ecosystems.

Engineered nanoparticles such as ZnO NPs, can easily become nano-contaminants during the production, utilization, and disposal process; and as these artificial particles do not occur naturally, living organisms may not have developed adequate coping mechanisms. The potential negative consequences of releasing engineered nanoparticles into the environment should be thoroughly evaluated, and future waste management strategies would do well to develop effective containment and disposal methods for ZnO NPs and other nanoparticles.

In conclusion, ZnO NPs were found to slow *B*. *subtilis* cell growth by prolonging FtsZ ring formation, and further prevented biofilm formation by reducing EPS production. ZnO was also confirmed by XANES and EXAFS spectra analysis to be present in *B*. *subtilis* cells cultivated with ZnO NPs. To our knowledge, this is the first study to show that ZnO NPs exert such effects on *B*. *subtilis*, and highlight the destructive potential of engineered nanoparticles to microbial organisms and soil ecosystems when released into nature.

## Supporting Information

S1 Fig Growth curves of *B*. *subtilis* wild-type cells grown in a minimal medium, supplemented with different concentrations of ZnO NPs. ZnO-NP concentrations are shown as -￭-: 0 ppm, -▲-: 10 ppm, -▼-: 25 ppm, -◆-: 50 ppm, and -◀-: 100 ppm.
(DOCX)

S2 Fig Growth curves of *B*. *subtilis* wild-type cells grown in LB medium that was pretreated with ZnO NPs for 30 h. ZnO-NP concentrations are shown as -￭-: 0 ppm, -◆-: 50 ppm, and -◀-: 100 ppm.
(DOCX)

S3 Fig Growth curves of *B*. *subtilis* wild-type cells grown in SSG medium, supplemented with different concentrations of ZnO NPs. ZnO-NP concentrations are shown as -￭-: 0 ppm, -●-: 5 ppm, -▲-: 10 ppm, -▼-: 25 ppm, -◆-: 50 ppm, and -◀-: 100 ppm.
(DOCX)

S4 Fig Growth curves of *B*. *subtilis sinR* mutant grown in SSG medium, supplemented with different concentrations of ZnO NPs. ZnO-NP concentrations are shown as -￭-: 0 ppm, -●-: 5 ppm, -▲-: 10 ppm, -▼-: 25 ppm, -◆-: 50 ppm, and -◀-: 100 ppm.
(DOCX)

S5 Fig Localization of FtsZ in wild-type cells grown under concentrations of 100 ppm of ZnO NPs in LB at 37°C. FtsZ was stained green; cell membranes were stained red; and DNA was stained blue. The closed yellow arrows indicate medial FtsZ rings.
(DOCX)

S1 Table Strains and plasmids used in this study. (DOCX)

S2 Table Fine structural parameters of *B*. *subtilis* cells treated with 100 ppm of ZnO-NPs, analyzed from EXAFS spectra. (DOCX)
