# Supplementary material for: ZnO Nanoparticles Affect Bacillus subtilis Cell Growth and Biofilm Formation
Source: PLoS One. 2015 Jun 3;10(6):e0128457. doi: 10.1371/journal.pone.0128457 (PMC4454653; doi:10.1371/journal.pone.0128457)
Supplement: S1 Fig — ZnO-NP concentrations are shown as -￭-: 0 ppm, -▲-: 10 ppm, -▼-: 25 ppm, -◆-: 50 ppm, and -◀-: 100 ppm. (DOCX) [file pone.0128457.s001.docx]

**S1 Fig. Growth curves of *B. subtilis* wild-type cells grown in a minimal medium, supplemented with different concentrations of ZnO NPs.** ZnO-NP concentrations are shown as: -￭- : 0 ppm, -▲-: 10 ppm, -▼-: 25 ppm, -◆-: 50 ppm, and -◀-: 100 ppm.
