# Supplementary material for: ZnO Nanoparticles Affect Bacillus subtilis Cell Growth and Biofilm Formation
Source: PLoS One. 2015 Jun 3;10(6):e0128457. doi: 10.1371/journal.pone.0128457 (PMC4454653; doi:10.1371/journal.pone.0128457)
Supplement: S2 Fig — ZnO-NP concentrations are shown as -￭-: 0 ppm, -◆-: 50 ppm, and -◀-: 100 ppm. (DOCX) [file pone.0128457.s002.docx]

**S2 Fig. Growth curves of *B. subtilis* wild-type cells grown in LB medium that was pretreated with ZnO NPs for 30 h.** ZnO-NP concentrations are shown as -￭- : 0 ppm, -◆-: 50 ppm, and -◀-: 100 ppm.
