# Supplementary material for: ZnO Nanoparticles Affect Bacillus subtilis Cell Growth and Biofilm Formation
Source: PLoS One. 2015 Jun 3;10(6):e0128457. doi: 10.1371/journal.pone.0128457 (PMC4454653; doi:10.1371/journal.pone.0128457)
Supplement: S5 Fig — FtsZ was stained green; cell membranes were stained red; and DNA was stained blue. The closed yellow arrows indicate medial FtsZ rings. (DOCX) [file pone.0128457.s005.docx]

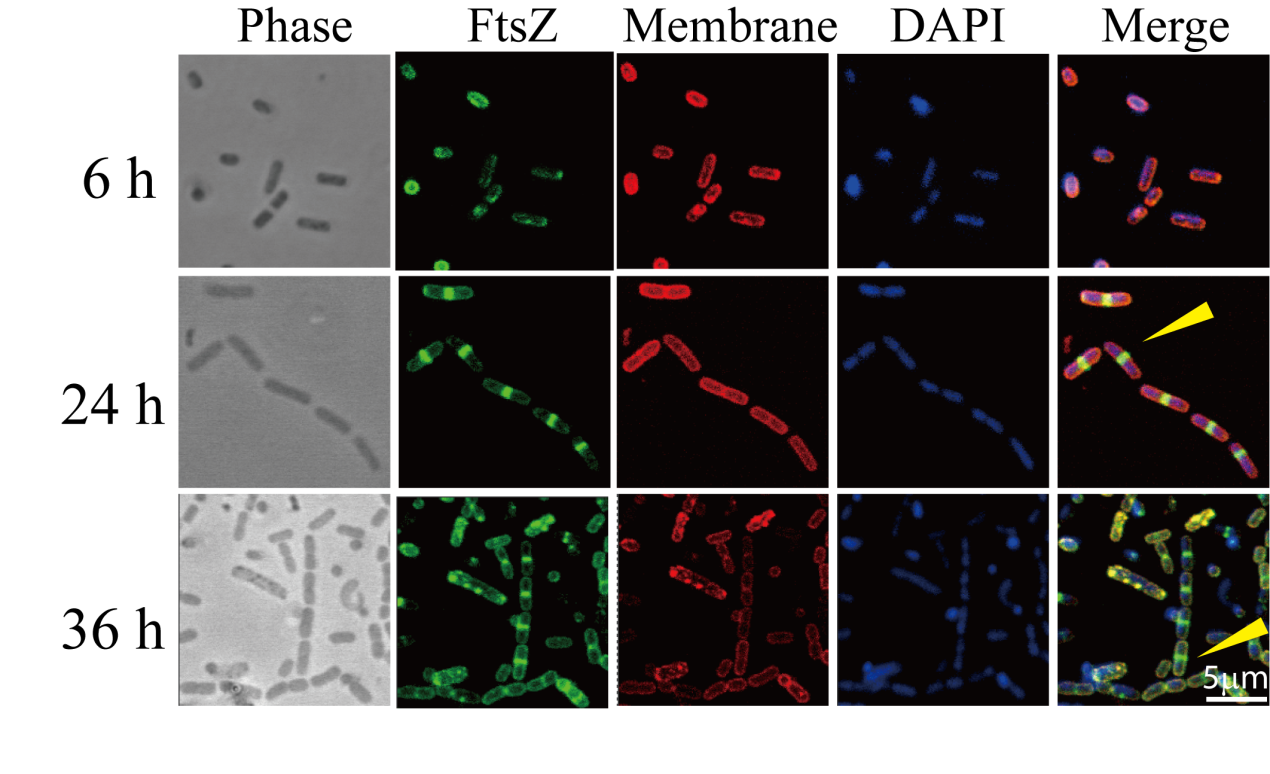
**S5 Fig. Localization of FtsZ in wild-type cells grown under concentrations of 100 ppm of ZnO NPs in LB at 37 °C.** FtsZ was stained green; cell membranes were stained red; and DNA was stained blue. The closed yellow arrows indicate medial FtsZ rings.
