# Supplementary material for: ZnO Nanoparticles Affect Bacillus subtilis Cell Growth and Biofilm Formation
Source: PLoS One. 2015 Jun 3;10(6):e0128457. doi: 10.1371/journal.pone.0128457 (PMC4454653; doi:10.1371/journal.pone.0128457)
Supplement: S1 Table — (DOCX) [file pone.0128457.s006.docx]

**S1 Table. Strains and plasmids used in this study.**

| Strains or plasmids | Description |
| --- | --- |
| 3610 | Undomesticated wild strain *B. subtilis* NCIB 3610 |
| DS859  DS646 | *sinR::kan*  *epsA-O*::*tet* |
| DS3337 | *sfp::mls* |
| DS3323 | *tasA::kan* |
| HS222 | *sinR::spec epsA-O::tet* |
| pHag-gfp | pHY300PLK with P*_hag_*-GFP *tet amp* |

All other strains are derivatives of 3610.
