# Supplementary material for: ZnO Nanoparticles Affect Bacillus subtilis Cell Growth and Biofilm Formation
Source: PLoS One. 2015 Jun 3;10(6):e0128457. doi: 10.1371/journal.pone.0128457 (PMC4454653; doi:10.1371/journal.pone.0128457)
Supplement: S2 Table — (DOCX) [file pone.0128457.s007.docx]

**S2 Table. Fine structural parameters of *B. subtilis* cells treated with 100 ppm of ZnO-NPs, analyzed from EXAFS spectra.**

| Shell (1^st^) | ^a^CN (± 0.05 Å) | ^b^R (Å) (± 0.01 Å) | ^c^Δ σ^2^(Å^2^) | R factor |
| --- | --- | --- | --- | --- |
| Zn-Zn | 59.4 | 2.21 | 0.038 | 0.006 |
| Zn-O | 3.9 | 2.05 | 0.013 | 0.002 |

^a^CN: Coordination number; ^b^R: Bond distance; ^c^σ: Debye-Waller factor.
